# Consequences and coping strategies six years after a subarachnoid hemorrhage – A qualitative study

**DOI:** 10.1371/journal.pone.0181006

**Published:** 2017-08-30

**Authors:** Hanna C. Persson, Karin Törnbom, Katharina S. Sunnerhagen, Marie Törnbom

**Affiliations:** Department of Clinical Neuroscience, Institute of Neuroscience and Physiology, Sahlgrenska Academy, University of Gothenburg, Gothenburg, Sweden; University of Pittsburgh, UNITED STATES

## Abstract

**Background:**

After a subarachnoid haemorrhage (SAH), continuing impairment is common and may impact the person’s life. There is a lack of knowledge regarding long-term consequences experienced.

**Purpose:**

To explore experiences of the care and rehabilitation as well as the consequences and strategies used to cope with everyday life six years post SAH.

**Methods:**

An explorative interview study with a qualitative design. Individual interviews, with open ended questions, using an interview guide were performed with sixteen participants (mean age 63, 8 men, 8 women) six years post SAH. Data was analyzed according to a descriptive thematic analysis, and themes were discovered inductively.

**Results:**

Two major themes from the analysis, both including four sub-themes, were identified; these themes were *consequences of the SAH* and *coping strategies*. Participants were grateful to have survived the SAH and most were satisfied with their acute medical care. If discharged directly from the neurosurgical unit participants can feel abandoned. In contrast, participants who were referred to a rehabilitation clinic felt supported and informed. Cognitive problems, such as impaired memory and mental fatigue, were reported as still present six years post SAH. Coping strategies were; receiving support from family, society, employers, or technical equipment. At work, talking to colleagues and to taking breaks were common. Participants described hiding their symptoms from employers and friends, as well as trying to continue doing tasks in the same manner as prior to the SAH. If this was not possible, some refrained from doing these tasks. They went through a mourning process, fear, and worries.

**Conclusions:**

Participants reported several long-term consequences which impacted on their daily lives post SAH, and different coping strategies were used to cope with these problems. Participants reported lack of awareness regarding the consequences of SAH and stressed the importance of structured multidisciplinary follow-ups, which mostly is missing.

## Introduction

People that survive a subarachnoid haemorrhage (SAH) may have cognitive impairment symptoms (memory problems or low executive function etc.) [1, 2], emotional complaints [3], depression [1], and/or fatigue [4]. Long-term visual memory and language difficulties have also been described [5]. The consequences of a SAH may have impact on a person’s life on quality of life [2] and people after SAH have a low rate of return to work [2]. Furthermore, psychological symptoms such as depression, anxiety and fatigue have shown to be strongly associated with decreased health related quality of life (HRQoL) [2, 4]. Three years after the SAH, 25% were shown to have post-traumatic stress disorder (PTSD) [6] which may also have an impact on HRQoL. A prior history of psychiatric disorder and the use of maladaptive coping strategies have been shown to be factors associated with depression/PTSD and reduced HRQoL [7]. A qualitative study of people one year post SAH [8] identified two patterns of perception of the recovery depending on whether participants had ongoing major depression or not [8]. People with depression had a more pessimistic perception of recovery, whereas those without depression had confidence in their recovery [8]. Several studies have described unmet needs after discharge from hospital and a lack of information of the cognitive and emotional consequences after SAH [8–11].

Coping after SAH might depend on different cognitive symptoms and life situations. Other factors influencing coping strategies are the individual personality traits as well as the choice of coping style [12]. According to the psychologists Lazarus and Folkman [13], coping can be defined as the sum of cognitive, emotional and behavioral efforts, which aim to handle particular stressors in various situations. Whether a situation is demanding and/or frustrating, or if the challenge is a problem is decided by the individual. Two significant examples of different coping styles were described [13]; problem-focused and emotion-focused strategies. Problem-focused strategies are used when conditions are deemed to be changeable, whereas emotion-focused strategies are used when conditions are thought to be not mutable and aim to regulate one´s emotions to tolerate or eliminate stress [13]. There is a broad field of articles about coping strategies related to different diagnoses. In a review article [12] of coping strategies and HRQoL after stroke, it was pointed out that well-being was related to the ability to use active coping strategies. Helpful strategies during recovery seemed to be information seeking, participation in rehabilitation, problem solving, and engagement in activities [14].

Even though the body of neuropsychological research of consequences after SAH is significant [15–17], less is known regarding patients´ subjective experiences and long-term strategies used to manage deficits in daily life post SAH. The purpose of this qualitative study was to explore experiences of three areas; the care and rehabilitation as well as the consequences and strategies used to cope with everyday life six years post SAH.

## Method

### Design

This study is an explorative interview study with a qualitative descriptive design, using an inductive driven thematic analysis [18]. The study follows the Helsinki declaration and was approved by the Regional Ethics Committee in Gothenburg (EPN) Sweden in June 5^th^ 2013 (Dnr: 400–13). Informed written consent was obtained from all participants except for one (aphasia and severe motor deficits); her next of kin consented (written) on her behalf. The study follows the COREQ criteria [19].

### Participants

Participants were included from the Extended Stroke Arm Longitudinal study at the University of Gothenburg, SALGOT-extended [20, 21]. The inclusion criteria were; ≥ 18 years of age, with a SAH between the 4^th^ of February 2009 until the 2^nd^ of December 2010, receiving care at the Sahlgrenska University Hospital in Gothenburg, Sweden, resident in the Gothenburg urban area (≤ 35 km from the hospital), able to speak Swedish and at least some participants should be of working age. The total number of eligible participants was 26. An invitation letter was sent to 20 persons according to the inclusion criteria (two persons had moved from the area and four were too ill to participate). The participants were contacted by phone and if they agreed to participate, a time an interview was planned. Of the 20 persons, 16 chose to participate, the mean age was 57 years old (min 45, max 76) at SAH, eight participants were male and eight were female.

Demographic characteristics from the acute hospital stay were collected from the medical charts. The initial neurologic clinical classification of the SAH was scored according to Hunt and Hess [22] (H&H, 1–5, lower is better). The acute medical treatment with either coilor conservative treatment and the overall status at discharge from acute care treatment at hospital was assessed according to the modified Rankin Scale [23] (mRS, 0–5 lower is better) from the medical charts. The number of participants that received one or more severe complications (such as re-bleeding, vasospasm, hydrocephalus, cardiac, cerebral infection) are presented in Table 1.

**Table 1:** Demographic and clinical characteristics of the participants (n = 16).

2009–2010 at SAH	n
Male/Female	8/8
Age, mean years (min-max)	57 (45–76)
Aneurysm/No aneurysm or unknown	11/5
Acute medical treatment, n	
*Coil (and stent)/Conservative*	11/5
Hunt and Hess, median (min-max)	2 (1–5)
Acute medical complication *(re-bleeding/vasospasm/hydrocephalus/cardiac/cerebral infection)*	
0 complication	4
1 complication	3
2 complications	5
≥3 complications	4
mRS at hospital discharge, median (min-max)	2.5 (0–5)
Admitted to rehabilitation unit at hospital yes/no	10/6

Abbreviations: mRS, modified Rankin Scale

### Data collection

All interviews were performed face-to-face using an interview guide with open ended questions: 1) What type of treatment (acute and rehabilitation) did you receive? 2) What type of consequences (such as cognitive and physical) do/did you have post SAH? 3) What strategies have you used to cope with the consequences of SAH in daily life? 4) What impact do the consequences post SAH have on your social function and in daily life? The interview guide was discussed among the authors prior to the interviews, and some questions were changed/added. The first interview was seen as a pilot for the interview guide, and the questions seemed to cover the research areas of interest. The interviews took place on average (mean average) six years (SD 0.5) post SAH. All interviews were performed during a 3 month period in 2015–2016 by one of the authors (MT) who has a PhD and >30 years’ experience as a social worker as well as a researcher using qualitative methods. The participants chose the time and place for their interview; six interviews were conducted in the participant’s home and ten at the university. During two interviews, the next of kin were present as a support because of the participant’s aphasia or memory gaps. Prior to the interviews participants were informed about the purpose of the study and briefly about the interviewers’ (MT) clinical and research experience and about the research team. This was followed by a short self-reported questionnaire of the participants´ current status regarding their housing, work and briefly of possible comorbidities, Table 2. Each interview lasted between 1–1.5 hours. All interviews except two were audio-recorded and transcribed verbatim by one of the authors (MT). In the interviews not audio-recorded, field notes were taken by the interviewer. Data saturation was achieved after 16 interviews. Transcribed interviews were not returned to the participants.

**Table 2:** Self-reported current status at the time of the interviews.

2015–2016, 6 years post SAH, (n = 16)	
Age at interview, mean years (min-max)	63 (50–83)
Married	14
Single or widow/widower	2
**Occupation**, n	
*Employed full time*	5
*Employed and Sick Leave*	1
*Employed and early retirement part time*	1
*Sick Leave*	1
*Sick Leave and early retirement*	1
*Early retirement full time*	1
*Retirement*	6
**Comorbidity,** multiple-choices is possible, n	
*Diabetes or hypertonia*	4
*Cancer*	3
*Mental illness*	1
*Anemia*	1
*Amyloidos*	1
*Rheumatism*	1
*Transplantation (kidney and liver)*	1
No reported comorbidity	6

### Data analysis

The data was analyzed according to thematic analysis described by Braun and Clarke as a flexible method [18]. The thematic analysis used in this study was inductive and can be referred to a realist/essentialist paradigm [18]. The text material was analyzed by two of the authors (HCP, MT), HCP has a PhD and is a registered physiotherapist with >15 years of clinical working with patients with neurological diagnoses. The analysis followed a six step procedure (Table 3). Step one was first analyzed separately by MT and HCP and then coordinated. The following steps (2–6) were analyzed coordinated and from step 4 the two authors, KT; MSSc, PhD student, a social worker with >5 years experiences of using qualitative methods and KSS; MD, PhD, Professor in Rehabilitation Medicine with >25 years of clinical and research experience in neurological diagnoses, contributed with suggestions and discussions. The analyses process moved back and forth between the different levels in the analysis and between whole and parts of the text to check that themes, codes and extracts from the text made sense. Authors discussed discrepancies until a consensus agreement was reached. The procedure to involve all four female authors (with different experiences and occupations, but no prior connection with participants) in the analysis process from step 4 contributes to ensure trustworthiness, credibility, reliability, and confirmability of the results. Furthermore, the in-depth interviews, not leaving a participant before all was communicated and understood, contributed to ensure relevant factors associated with experiences of SAH were collected. The recommended 15-point checklist of criteria for good thematic analysis [18] including transcription, coding, analysis, and fulfilling the report was followed.

**Table 3:** The six steps for the thematic analysis according to Braun and Clarke.

Step	Content
1	Familiarization with data: transcribing, reading, re-reading, taking notes
2	Generation of initial codes: coding interesting feature across the entire data set
3	Searching for themes: collecting codes into potential themes, gathering all data relevant to each potential theme
4	Reviewing themes: generate a thematic “map” of the analysis and check the themes relevance in relation to coded extracts (level 1) and entire data (Level 2)
5	Defining and naming themes; Ongoing analysis to refine the specifics of each theme, and the overall story the analysis tells, generating clear definitions and names for each theme
6	Producing the report; The final opportunity for analysis: selection of vivid, compelling extract examples, final analysis of selected extracts, relation back of the analysis to the research question and literature, producing a report of the analysis

## Results

**Fig 1:**
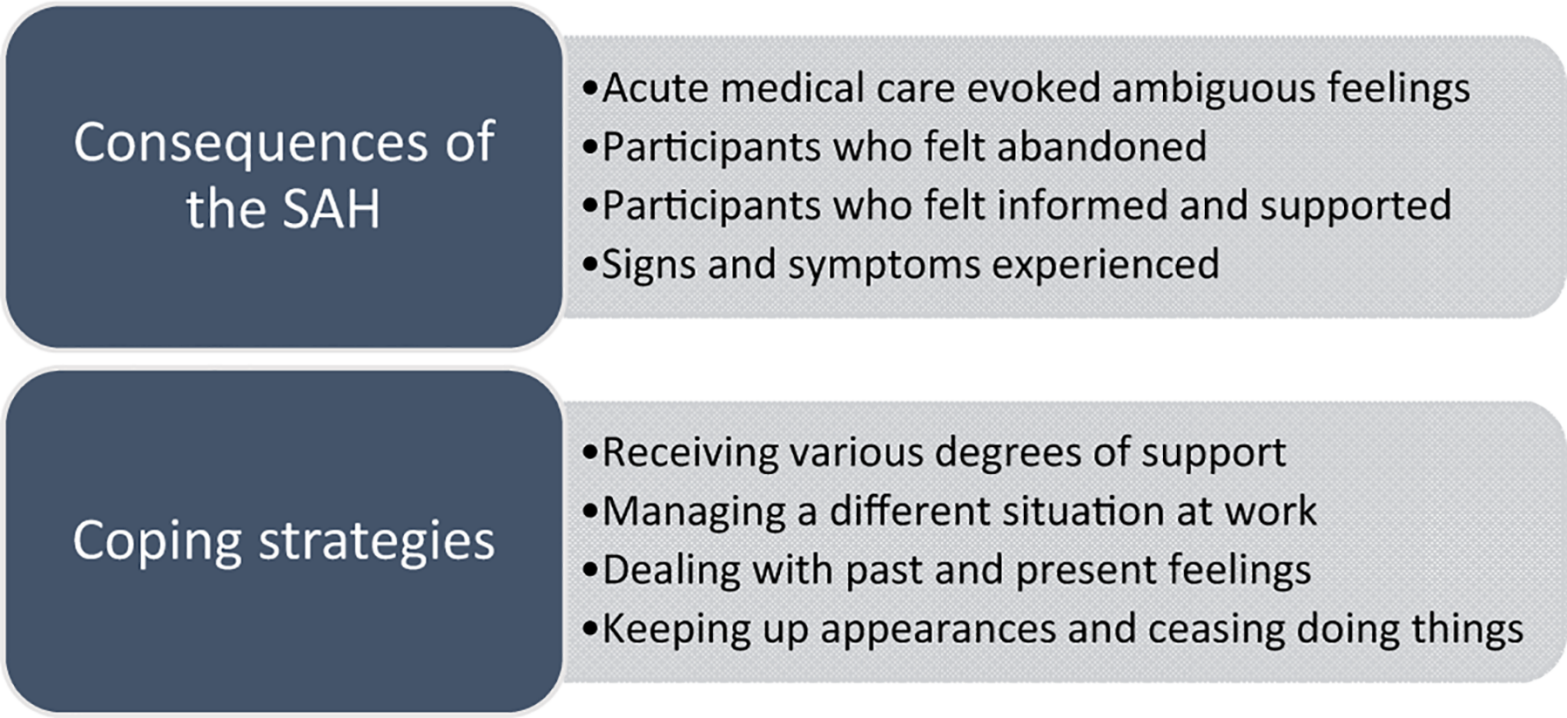
Themes and sub-themes in the current study.

Two major themes were identified; c*onsequences of the SAH* and c*oping strategies*, both with four sub-themes (Fig 1) and illustrated with quotes from the participants. The main theme *consequences of the SAH* comprises description of the acute treatment, rehabilitation and perceived symptoms. The second main theme, c*oping strategies*, describes participants coping strategies used since the SAH mainly focused on long-term coping strategies, used at the time of the interview.

## Consequences of the SAH

### Acute medical care evoked ambiguous feelings

Participants articulated a gratitude for having survived the SAH, and in general, they thought very highly of the acute medical surgery and services. After the first medical interventions, participants described that their disease condition varied and for some, medical complications worsened the course of their illness. However, in the chaotic unknown situation at the hospital, some participants felt that they were left alone and missed being cared for, or felt that research was considered more important than the patients were. On the other hand, they also expressed positive attitudes about the healthcare professionals.

*“You’re so grateful yeah*? *There was a nurse who was so lovely*, *so adorable who said you had to eat something*. *After I came to (after being put under) I said I really fancied an orange… and she brought me one and I asked “did you guys have oranges*?*” and she said “No*, *this one was mine”*. *I will never forget that… “No*, *this one was mine”*. *Amazing staff*.*”* (Participant 8)

When looking back at their emergency hospital stay, participants reported memory gaps and feelings of a distorted reality, which they found hard to come to terms with. Furthermore, some worried that their next of kin had had a hard time during the course of their acute illness.

### Participants who felt abandoned

Participants who were discharged home after a short period at the neurosurgical unit felt lonely, as well as abandoned and worried. They expressed that their cognitive symptoms became evident in everyday life, and most of them did not understand why they had these symptoms. They expressed that they did not have enough knowledge about the consequences after a SAH, and wished they had received more information about the course of the illness along with possible cognitive consequences before discharge.

*“It’s like you just were sent home from the hospital*, *no-one cared after that…*.*go home and get back to work*, *pretty much*, *it wasn’t anything that bad…*. *I was so unwell that when my husband came to get me*, *he had to guide me and help me walk because I had no sense of balance*, *I saw double…all the windows were like long rows*, *rows and rows of floating windows*.*”* (Participant 15)

Participants described different experiences from follow-ups; some had X-ray screening for new aneurysms, whereas others did not get a follow-up meeting and had to contact their primary health care facilities themselves, which also made them feel abandoned. Some expressed that they would have needed someone with medical expertise and/or a counsellor to talk to after discharge.

*“I felt like the treatment at the hospital*, *in the two weeks I was there*, *was great…so I was a bit let down afterwards…*.*I was very well taken care of at the hospital and then after I was discharged it was like it was all on me…it felt like I was completely cut loose…*.*you can look after yourself now…”*(Participant 1)

### Participants who felt informed and supported

Participants who were referred to the rehabilitation clinic after their acute care described that they felt informed about the symptoms that appeared after the SAH. They also felt that healthcare professionals acknowledged their individual symptoms and experiences. Participants expressed appreciation and gratitude towards the healthcare professionals, both their skills and their availability. In some cases the expertise of the physiotherapists was especially valued.

*“I had been bedridden for more than two months*, *so my muscles had wasted away to the point that if I sat down I couldn’t get back up again and stuff like that*. *So the biggest thing was the physiotherapist*, *the physiotherapist is the one I remember the most*. *She did an amazing job*.*”* (Participant 10)

Most of them said that they felt sufficiently rehabilitated at discharge from the rehabilitation clinic, but some wished for a longer rehabilitation period and felt fragile. After discharge, participants reported that they were referred to different health facilities, if needed. Some would have liked follow-up appointment/s after discharge and this was not offered. Everyone appreciated being able to receive care at the rehabilitation clinic.

### Signs and symptoms experienced

Reported experienced symptoms such as aggressive isolated outbursts, incomprehensible speech and visual deficit/s; such as hallucinations, loss of sight and/or problems with double vision were only experienced during the first months post SAH. Some worried about relapse, but most had been convinced by doctors not to worry about eventual relapses.

However, several cognitive symptoms were reported six years after SAH; impaired memory, mental fatigue, difficulties with concentration, lack of time perspective, sensitivity to sound and light, lack of executive function and difficulties in reading and understanding the written word. Psychological consequences such as; altered personality, anxiety, depression or panic attacks were also reported.

…*my perception of time is defective*, *purely intellectually I know what happened last week but I don't have sense time so things that happened four years ago are the same as if they had happened last week*… (Participant 3)

The most predominant physical deficits reported by the participants were hemiparesis, difficulties with balance and/or dizziness. All symptoms were reported to be less pronounced at the time of the interviews compared with the beginning of the rehabilitation phase. A few had experienced a sudden improvement in symptoms while others said that symptoms still fluctuated from time to time.

## Coping strategies

The second main theme *coping strategies* with the four sub-themes are shown in Fig 1. Participants felt that life had changed after the SAH and that they needed new coping strategies to deal with everyday life. Described coping strategies had been used more or less explicitly since the SAH and had varied over time.

### Receiving various degrees of support

Shortly after the SAH participants said they were inspired by the rehabilitation clinic and/or primary health care clinic and viewed physical activity as something very important. Some had started to practice physical exercise while others expressed an ambition to start. Some had stopped smoking, lost weight or/and thought about what food they ate. These factors together resulted in a fundamental change of lifestyle for some participants.

“*But afterwards…I’ve talked to one of the doctors at the (primary health) clinic*, *you could say I’ve changed my lifestyle a bit…I’ve changed how I eat a lot…I’ve started moving more*.” (Participant 10)

They also expressed being supported by their spouse in reaching goals in physical rehabilitation. Due to physical impairments, two couples moved to a single-story house (summer houses) during their rehabilitation period. Other coping strategies described were; applying for in home care and/or transport available for people with disabilities, or using the tax-reduced home services available in Sweden. Some participants reported that they had lost their driver´s licences, and as part of regaining their licence, participants were assessed at a mobility center.

Some participants expressed that in the early phase they would have needed increased support from their primary health-care physician, who did not have sufficient knowledge of SAH. A coping strategy to handle this was to gather information from other sources. Participants also reported that due to policy changes from the government, the number of days they were able to take of sick leave were affected, and that a doctor might be reluctant to prolong their sick leave certificate. As a result of these circumstances, some participants said that they needed more days away from work on sick leave than they were granted from the authorities.

Participants reported long-term strategies to cope with cognitive deficits such as receiving help from their family members with some activities at home as well as cognitive support, for example to remember things for them. The received support was dependent on the spouses’ health situation, and different ability to support and helping each other was reported.

*“Because I said to…*.*the doctor that I think this is going to end in divorce if I don’t get help*, *because I was depressed…It is obvious that it wasn’t fun*, *he (my husband) also became sad (he’s also sick) and angry…and then I became even sadder*.*”* (Participant 11)

Due to the SAH and in ability to work, the economic consequences were more evident for persons who lived alone.

*“I was alone then*, *I didn’t have a partner and my wages decreased*, *initially I was on sick leave and then I got the disability pension …and there was a big difference*, *many many thousands (of Swedish kronor)…”* (Participant 13)

However, most thought that the economic consequences after the SAH were not too bad. Separate housing due to severe disability post SAH led to increased expenses for some couples. One strategy to manage strained economic circumstances could be saving money by asking a relative to cook meals.

In order to cope with mental fatigue participants described that they had attended mindfulness courses. Others had tried to participate in groups of patients with stroke, in order to share experiences. However, experiences of others with stroke were described as painful to listen to. Some said in order to learn more about the consequences of the SAH, they read news articles about it.

Participants also reported technical support. When they lacked the ability to read books, one strategy was to listen to audio books. Using a computer or mobile phone to help them have a greater grasp on time was one way they coped.

*“So I use my computer as a tool and have done well doing that*..*every day I have a timetable of what I am going to do*. *I go and look at my computer many times a day*. *And I am very pleased at being able to do it that way*..*If i didn't have the timetable on my computer it would have been complicated*..*and then I use a timer on my phone*, *I put a timer on when I’m doing different things*..*It is my saviour*.*”* (Participant 3)

### Managing a different situation at work

Of those returning to work, the experience of the involvement from their employer varied from being neglected to receiving sufficient support. Some reported that their employer did not ask what they might need after the SAH. On the other hand, one participant described that their employer sent them home when they were very tired in order to rest, and another one arranged a chauffeur to help one participant who could not drive. However, the importance of support by colleagues was stressed by everyone, both on an emotional and a practical level.

*“So she (my colleague) helped me a lot too*,*…I was supposed to count*, *but I couldn’t*, *it was always wrong*, *and especially after she (my colleague) had finished for the day*, *it really didn’t work…*.*”* (Participant 13)

One strategy used when returning to work was to start increasing working hours gradually, supported by the governmental insurance office. Some, who still suffered from consequences of the SAH after the first period of sick-leave, chose to reduce their working hours without economic compensation.

Some participants, not admitted to a rehabilitation clinic, reported that they returned to work after a shorter period of sick-leave or even before their sick-leave was ended. They returned to their former work place, and reported that they tried to adapt to their former working tasks immediately and found it difficult. In this group, the most often mentioned coping strategies in order to deal with work-related difficulties were; leaving a task, resting or talking to colleagues, and then when they returned to the tasks it was easier to concentrate. One participant said that he was so tired that he slept during his lunch break in the first few weeks.

*“…yeah*, *you get tired…crazy tired*…*they (my colleagues) laughed and said XX is sleeping in the middle of his break*…*in the lunchroom*…*but it was really lovely (to be able to sleep)…”* (Participant 8)

Some of those who had been admitted to a rehabilitation clinic were also referred to a vocational rehabilitation center. They thought this improved their work capacity and they also reported that the participants of the vocational rehabilitation group bonded with, and supported, each other.

*“The first time I made it (a chair) it took at least 14 days to manage*, *but then later*, *after vocational rehabilitation*, *I did another one*, *and it took just a day*…*We ended up being a really nice group there (at the vocational rehabilitation center) too*. *I and a patient go once every three months to the vocational rehab center and take it easy and do some curling…”*. (Participant 16)

Participants described adaptations and strategies that they used to handle new conditions in the workplace. For example; sitting at their desk due to balance problems, having to take notes in order to remember things or change from nightshift to dayshift because of tiredness or depression. Some also reported that they had to change work assignments due to cognitive or/and physical limitations.

### Dealing with past and present feelings

Most participants who still experienced problems at the time of the interview had learnt to live with their current situation and had adapted life according to the consequences and symptoms of the SAH. One strategy mentioned by several participants was to concentrate on having a positive outlook and ignoring negative thoughts.

Some described that they worried a lot about their memory gaps and some thought that they had developed dementia. One participant was even tested for dementia, with a negative result, in trying to understand why he/she had memory gaps. Some participants reported that they had suffered from depression or experienced panic attacks/anxiety and some used anti-depressants to manage their daily lives. Participants explained that their loss of physical functions had sometimes caused frustration or an emotional mourning process. Several said they were distressed over their inability to read and understand what they had read.

*“Whereas I think I have gotten worse at reading*, *I have enjoyed reading but it is slow going*..*I start and then put the book down on the bedside table*, *I think that’s sad…it’s a loss*.*”* (Participant 14)

The fear of having a relapse hindered some participants from performing physical activities.

*“Above all*, *if you were going to lift weights and really go at it*..*the first 2–3 years I would never have done it*, *I was worried it would just go ‘poof*!*’ Even now it is one of the reasons that I have dragged me feet with it (physical exercise)…”* (Participant 7)

### Keeping up appearances and ceasing doing things

In order to keep up appearances, participants reported that they hid their symptoms and sometimes avoided revealing them. Some said, regardless of their age, that they had explained to themselves that their vague cognitive consequences of the SAH could be interpreted as age-related.

*“I think it is more difficult to remember names now*, *but I don’t know if it is because I am getting older or it is the bleed that is behind it*.*”* (Participant 1)

Some expressed that they hid symptoms, especially in front of their employers and colleagues, to be able to stay with the same work team and the same work tasks. They said that this hiding strategy was used to avoid special attention from colleagues and employers, they wanted to be regarded as perfectly normal and capable of managing their work tasks.

*“I really wanted to stay there*, *so I hid problems and didn’t talk about how hard it was for me and that was maybe not a great idea…it was really really valuable for me to be able to stay (in that job)*..*so I didn’t want to end up in the position where people would have to be careful around me or change anything because I had something wrong with me or whatever*, *you want to minimise that somehow*, *so that everything could be…normal*.*”* (Participant 7)

*“It was something I didn’t want to mention at all at work*, *but I’m not as sharp as I used to be*..*and the doctors said that to me too*, *that I would be a little slower*..*”* (Participant 1)

Another hiding strategy mentioned was interpreting a work situation as stressful, instead of admitting cognitive impairments. Participants also described hiding problems when meeting friends and relatives. Problems with communication, such as formulating words, remembering or thinking fast hindered participants in speaking freely when meeting friends, family members or colleagues. It was more convenient to hide these problems and let others talk and concentrate on listening.

*“I have good friends*, *so I maybe rest a little bit before they come over and then I’m able to hang out with them then and don’t have to be so involved later*..*and you can be quiet*, *you don’t have to say so much*. *you can just let them talk*..*people like that…”*(Participant 3)

When the consequences of the SAH were too pronounced, refraining from doing things was another reported strategy. Some participants said that they were too tired to meet friends and needed all their spare time to rest. When their cognitive problems were overwhelming at work, taking early retirement was seen as one way out. Others described that they had lost their ability to multitask and had to really concentrate to even manage one task at a time. Some participants reported that they had stopped walking on uneven ground because of difficulties with balance and chose to walk on flat ground.

**Fig 2:**
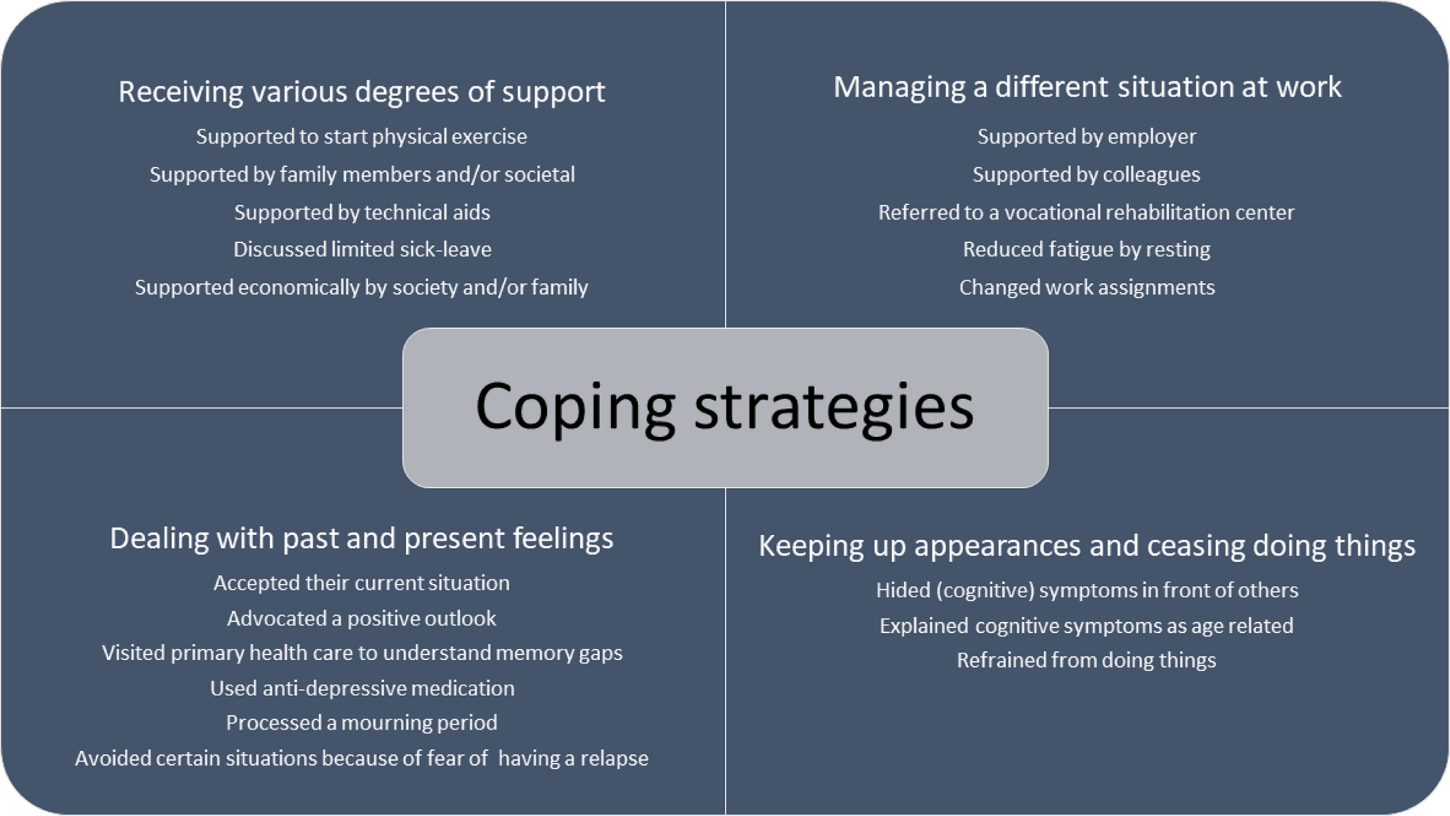
Described coping strategies to deal with life six years after SAH.

Reported coping strategies have been summarized in Fig 2.

## Discussion

The two major themes from the analysis were *consequences of the SAH* and *coping strategies*; participants described signs and symptoms that were still present six years post SAH, and different strategies were used to cope with life. Participants were grateful to be alive, but described that they had not been fully prepared for the potential consequences the SAH had on their life, which has also been reported from patients with stroke [14]. Early during the analysis process, two patterns among the participants were seen. Participants discharged home from the neurosurgical unit and those that were referred to a rehabilitation unit after the neurosurgical unit had different experiences and feelings. This pattern seemed to be more important than ongoing depression or PTSD, which earlier has been shown to have an impact on experiences and coping strategies following SAH [2, 4, 6–8].

Participants reported cognitive symptoms seemed to present persistent difficulties life, also shown previously [24]. Impaired memory, executive functions, and attention are cognitive deficits that have been shown to be common after SAH [2, 5, 10]. Some participants reported that their cognitive symptoms had resolved, while others struggled with these vague or more pronounced problems daily. The ability or lack thereof to handle cognitive (as well as physical) deficits may continue long time post SAH, and may also have an impact on improved QoL more than a decade after a SAH [25].

Participants that were discharged directly from neurosurgical unit had to manage their own recovery and thought it was hard to find appropriate support and to deal with rehabilitation. After stroke, the drive to return to meaningful activities has been shown to be strong, and people want to return to their previous lives [26]. From the present study, it was obvious that participants tried to manage and to perform acceptably at work and they started to work without complete information about the cognitive symptoms they experienced. Working in and of itself was exhausting and dealing with different cognitive symptoms at the same time made it even more difficult. Strategies used to cope with the cognitive consequences were to step away from their work, and after these breakes (resting or talking to colleagues), they felt it was easier to concentrate on work tasks. Another strategy that aggravated tiredness/stress, and made the phase after SAH at work more difficult, was deliberately hiding their symptoms in order to retain their position in the same work team or/and work tasks.

Even though the participants that were discharged to a rehabilitation clinic before coming home also reported perceived cognitive symptoms, they felt informed and supported by competent healthcare professionals. Similar results have been shown in another study of stroke survivors where a period in rehabilitation setting assisted people with stroke to regain control of their life [27]. Discharge from the rehabilitation clinic was described as well organized, based on the individual functional level and participants seemed to be better prepared for life after SAH.

In the present study task-, emotional- and avoidance- coping strategies [13] were seen, and the most prominent category was problem-focused strategies. Problem-focused strategies such as support from health care workers, municipality services, employers and/or family in practical matters were described and some reported the use of technical support as invaluable. The coping strategies used in this study such as information seeking, participation in rehabilitation, problem solving and engagement in activities are in accordance with another study [14]. Participants described emotional support from family, employers or colleagues. Inner struggles experienced were worries, mourning processes or fear. Some participants had reached acceptance and a positive outlook, and this was shown to be important to manage their new situation successfully [12, 28]. Some participants changed their life style and revised their values and priorities, this is in accordance with another study [29]. Avoidance strategies, that is hiding symptoms, were more pronounced at work than in other social contexts.

Areas identified for potential future study should be attempts to address the following questions; If people do not openly talk about their cognitive symptoms at work, will the work tasks be performed correctly and on time? Will a person´s behaviour confuse their co-workers and make the performance of a work team more difficult? Participants in the present study missed follow-ups, predominantly in the group that was discharged directly home from the neurosurgical unit. A lack of knowledge about the consequences after SAH might lead to persons speculating about irrelevant causes of cognitive symptoms and this could be avoided with structured follow-up appointments. For example, several of the participants interviewed argued that their cognitive symptoms probably were a result of ageing and not primarily consequences of SAH, although the participant that argued this most fervently was middle-aged. The need of follow-up visits after discharge from hospital has been pointed out previously [8–11] and a multidisciplinary out-patient clinic dedicated to persons with SAH and their families has been suggested [10, 24].

### Strengths, limitations and future perspectives

A strength of the present study was the use of a qualitative explorative design with an inductive driven analysis. To our knowledge, this is the first qualitative study of experiences of SAH regarding consequences and coping strategies after SAH. Another strength was the use of the thematic analysis method [18] which made it possible to discover the most important themes and strategies of the material. A semi-structured guide was used to ensure that the interviewer concentrated on specific topics related to the purpose of the study. A limitation was that the participants´ descriptions are related to experiences of the Swedish societal context and this needs to be considered when the knowledge is transferred to other cultural contexts. Further research could investigate what impact cognitive rehabilitation may have on strategies to manage life after SAH; a mixed-method design could combine patient perspective with quantitate data including cognitive assessment and questionnaires.

### Conclusion

After six years, participants experienced cognitive symptoms to a varied extent. Coping strategies used were practical and emotional support from: health care workers, municipality services, employers and family. Inner struggles focused on worries, mourning processes or fear. Some reached acceptance and a positive outlook. Hiding cognitive symptoms in different social contexts seemed relevant from some participants´ perspective. Participants also experienced a lack of awareness regarding possible consequences of SAH, especially if discharged directly from acute neurosurgical unit, and stressed the importance of follow-up appointments.

### Clinical implications

This study identified different experiences depending on whether a person was discharged from neurosurgical unit or from a rehabilitation regardless of the severity of the SAH. As the hospital stay after a SAH can be short, patients need improved information surrounding possible cognitive consequences. Follow-up visits at out-patient clinics or information sessions for groups of patients with SAH, a webpage with information to which patients could be referred or sending brochures about consequences post SAH could help these persons in their new life situation.
